# The Treatment of Proximate Cavities

**Published:** 1891-02

**Authors:** George Howe Winkler


AMERICAN JOURNAL

—OF—

DENTAL SCIENCE.

VOL. XXIV. BALTIMORE, FEBRUARY, 1891. NO. 10.

ARTICLE I.

### THE TREATMENT OF PROXIMATE CAVITIES.

BY GEORGE HOWE WINKLER, M. D., D. D. S.

[Read before the New York Odontological Society, Oct. 21, 1890.]

In offering this paper upon the subject of the treatment of proximate surfaces of teeth before the Odontological Society, I fully comprehend that the fundamental principles therein contained are so thoroughly understood that I can only hope to excite interest by confining myself almost entirely to the details of my own methods, and in doing so I shall be as concise as possible. In the formation of proximal cavities in the incisors and cuspids, I generally conform to a plan somewhat similar to that advocated by Dr. Arthur, of forming V-shaped or self-cleansing spaces by cutting away the posterior proximal surfaces of the teeth to be operated upon and inserting what Dr. George S. Allan, in his recent paper upon this subject, named "face-fillings." Slight grooves should be made in the cervical walls, and slight under-cuts opposite where these cavities do not encroach upon or involve the cutting edges, in which cases the under-cuts should be made along the incisive edges, the front plate of enamel being left intact. This is my rule, and, while I deviate from it at times, owing to the exigencies of special cases, I consider it the best means of protecting these teeth for long periods of time from further decay. None of the objections urged to the cutting away of molars or bicuspids in Arthur's method apply here, and by following this plan a display of gold is avoided, which is disfiguring, and the duty of that avoidance is in my estimation second only to the duty devolving in the preservation of the teeth themselves. Naturally, if a tooth is broken away, I restore the contour by building up with gold, or adjust a porcelain crown. I am a staunch advocate of preserving or restoring the contour of bicuspids and molars; in simple cavities, where space offers, filling flush with the tooth; in others where decay has destroyed the grinding surface, or it is necessary to approach a cavity by cutting away sound tooth-substance, the contour should invariably be restored. It is better that the filling should be top-heavy rather than provided with insufficient material. In that case the anchorage is extended more completely over the grinding surface, as is advocated by Dr. Parmly Brown. Usually it is better in proximate teeth to enter the carious cavity by cutting through the grinding surface; but in cases where decay has taken place near the gingival margins, with a strong body of enamel and dentine between it and the grinding surface, it is often advisable to drill into the decay through the buccal surface, running a tunnell, as it were, along the approximal, parallel with the decay, and so reach and arrest it. In the preparation of cavities the line of cleavage should generally be followed as nearly as possible, the edges of the cavity being very slightly rounded so as to allow the filling to flap over them rather than the reverse. Retaining pits should never be used in these locations, nor, in fact, in any others except where cast fillings are employed.

They are unnecessary in my method of operating. In their place slight grooves should be made in the cervical wall which cuts off the nutriment from so thin a layer of dentine and enamel rods that the overlapping filling at the bevelled edge of the cavity covers and protects a large proportion of them. Strong anchorage should be secured across the crowns, where the filling is made through the grinding surface, and slight under-cuts above and below where drifts are sent in from the buccal sides of the teeth. I fill all simple proximal cavities except in rare cases with non-cohesive foil, and never begin a contour filling with cohesive gold. Whenever it is necessary to contour a tooth, non-cohesive foil should be packed against the cervical wall, and the proximal cavity should be filled with it to within about a line of the grinding surface, where the palatine or lingual and buccal walls are intact, and as far as possible where one or both of these walls may be wanting. Cohesive foil is then welded to the already thoroughly condensed mass and the contour and grinding surface restored. In very soft bicuspids and molars, or where the cavity of decay has somewhat encroached upon the pulp, tin-foil should be used in place of non-cohesive gold. In lower molars, where the destruction of tooth-substance has extended quite to or a little beneath the gum, it is frequently advantageous to fill the cavity partly up with copper amalgam and at a subsequent sitting to finish and polish this filling and then contour the tooth with gold. Dr. Gordon White, in the past year, has introduced copper foil for plugging against the cervical walls, and asserts, "It does not look pretty, but it saves the teeth." In contouring upper teeth I never use the rubber dam, because in my method of operating it is not needed, instead of which I use separators, wedges, matrices of various forms, and napkins, which I change during the operation as they dampen. In the treatment of proximate surfaces, where the progress of decay has nearly or quite exposed the pulp, it should be immediately extirpated, the roots filled, and the crown proceeded with as before. In the case of young persons, where the functions of the pulp have not been fulfilled, I cap each pulp with a mat of tin-foil and insert temporary fillings, which are frequently examined and renewed when necessary. In time, the patient having arrived at proper age, the temporary fillings may be replaced by permanent ones, the tin mats being still retained, or the pulps may be extirpated. To one who has never operated with tin-foil, or non-cohesive gold-foil in combination with cohesive, it would be impossible to satisfactorily explain the great value of this method. At least two-thirds of each cavity can be filled with the greatest facility and in the shortest time. The danger of bruising or crumbling the walls is avoided by the presence always of a comparatively thick pad of gold between the plugger and the fragile tooth. The tooth is relieved of the long-continued malleting required in an all-cohesive foil filling, both patient and operator are saved great fatigue, and, in the case of the former, sometimes utter exhaustion. A most perfect hermetic sealing of the cavity is also secured, and a surface for the final finishing is presented sufficiently durable for all practical purposes, and yet soft enough to enable the operator to readily make a perfect finish at the cervical wall. There is no danger of small flake-like particles overlapping the surface of the tooth beyond the filling and probably causing a renewal of decay. A remarkable strength of union is obtained between these foils by beginning the cohesive-foil work upon the thoroughly condensed non-cohesive with small pieces of gold hot from the lamp. Each piece of gold then is freshly annealed as used, and every instrument is warmed to drive off the condensed moisture of the air. The instruments with which I pack gold are all smooth. In plugging with non-cohesive foil I work on the mechanical principle of a dovetailed mortice, and with cohesive foil I depend entirely upon its property of cohesion; never, under any circumstances, either depending upon or desiring the interdigitations made by serrated or sharp-pointed instruments.

Such, gentlemen, is a brief statement of my method of treatment of proximal surfaces, and while I am aware that the tenets I hold are opposed by many of our profession, I believe them to be the best. Non-cohesive and cohesive foil operations are distinct arts, both of which are beautiful in their perfection, but the intelligent combination of them must, in my opinion, ever remain more valuable than either. There exists in our profession, as in other medical specialties, a certain amount of scepticism in the presence of propositions which are contrary to pet theories and tried practices, and I expect that at least some portions of the above creed will be opposed with vigor. With thanks, gentlemen, for your kind attention, I have finished.—*International Dental Journal.*

